# CNS active O-linked glycopeptides

**DOI:** 10.3389/fchem.2015.00040

**Published:** 2015-06-24

**Authors:** Evan M. Jones, Robin Polt

**Affiliations:** Robin Polt Lab, Department of Chemistry and Biochemistry, The University of ArizonaTucson, AZ, USA

**Keywords:** glycopeptide, peptide, enkephalin, CNS, blood-brain barrier, analgesia

## Abstract

Naturally occurring glycopeptides and glycoproteins play important roles in biological processes. Glycosylation is one of the most common post-translational modifications *in vivo*. Glycopeptides are involved in cell signaling and sorting, providing cell surface markers for recognition. From the drug design and synthesis perspective, modification of a peptide through glycosylation results in increased bioavailability and bioactivity of glycopeptides in living systems with negligible toxicity of degradation products. Glycopeptide synthesis can be accomplished through incorporation of a glycosylated amino acid in solid phase peptide synthesis (SPPS) to form the desired peptide, or via incorporation of sugar-amino acid moieties. Additionally, research indicates that glycosylation increases penetration of the blood-brain barrier (BBB) by peptides, which may lead to novel therapeutics for neurological disorders. Recent applications of glycopeptides have focused on the *in vivo* central nervous system (CNS) effects after peripheral administration of centrally active peptides modified with various carbohydrates.

## Introduction

Endogenously derived peptides are attractive as a source of drugs due to their potent and highly selective interactions with G-protein coupled receptors (GPCRs), combined with their very low levels of toxicity (Kaspar and Reichert, 2013; Fosgerau and Hoffmann, 2015). The presence of proteolytic degradation pathways for peptides suggests that “off target” effects produced by metabolites will be minimal. In contrast, classical “small molecule” drugs are frequently promiscuous, leading to unpredicted off-target interactions as well as toxic metabolites (Rao and Mohamed, 2011). These same proteolysis pathways also represent a challenge since many peptides exhibit serum half-lives of only a few minutes (Fosgerau and Hoffmann, 2015). In addition, because drug development generally revolves around the synthesis and modification of small, organic molecules, peptide drugs do not fit within most of the widely accepted “rules” governing modulation of distribution, metabolism, and pharmacokinetic effects (DMPK), and therefore classical drug design strategies are ineffective (Lipinski et al., 2001). Peptides also necessitate careful handling and optimization of a separate set of challenges, including membrane transport, and the previously noted ease of metabolism and degradation. For peptide drug candidates targeted to the central nervous system (CNS), crossing the blood-brain barrier (BBB) is a challenge due to tight junctions, and is home to several peptidases (Egleton et al., 2000). Therefore, modifications to peptides to alter their DMPK have been widely explored (Diao and Meibohm, 2013). One modification that has shown much promise in recent years is glycosylation: appending a carbohydrate moiety along the peptide backbone to form glycopeptides. This is traditionally performed through O-linked, N-linked, C-linked, and more recently, S-linked (Zhu et al., 2003; Whalen and Halcomb, 2004) glycoside formation, as well as the synthesis and integration of “*sugar amino acids*” into peptides (Chakraborty et al., 1998; Prasad et al., 2005; Simone et al., 2005; Risseeuw et al., 2013). While examples of each can be found in nature and in the laboratory, this review will focus on O-linked glycopeptides. Alternate modifications including phosphorylation of peptides (Yeomans et al., 2011) and development of cyclic peptides have also been shown improve their drug-like properties, although those are not the focus of this review.

Glycopeptides play a wide range of roles in the regulation of many biochemical functions (Taylor, 1998). Notably, these include cell signaling and immune system response (Rudd et al., 2001). Additional post-translational modifications can provide both N-linked and O-linked glycoproteins through further elaboration by glycosyltransferases (Penno et al., 1989; Hennet, 2002; Cohen and Varki, 2014). In medicine, glycopeptide antibiotics such as vancomycin and bleomycin A_2_ are effective treatments of methicillin-resistant *Staphylococcus aureus* (MRSA) and Hodgkin's lymphoma, respectively.

In some cases however, glycopeptides can produce adverse effects. For instance, Rudd and coworkers described an overexpression of a glycoprotein, MUC1, in patients with breast cancer (Storr et al., 2008). They hypothesized that the increased presence of sugars on epithelial cell surfaces, from glycoproteins and glycolipids, was responsible for decreased immune responses. While it was noted that different types of breast cancer each had different aberrations [for instance, purified MUC1 from T47D breast cancer cell lines has primarily core 1 type *O*-glycans, while MUC1 from MCF-7 is composed of core 2 type *O*-glycans (Muller and Hanisch, 2002)], all breast cancer cell lines displayed an increased number of carbohydrates per glycoprotein when compared with healthy cells.

## Benefits and disadvantages of carbohydrate incorporation

The benefits of glycosylation toward increasing drug-like activity of peptides are manifold: in general carbohydrates improve peptide DMPK within living systems, all without introducing toxic compounds or metabolites.

### Stability

Glycosylation of peptides can reduce their susceptibility to enzymatic degradation. Enzyme binding pockets in proteases need unhindered access to the target site on the peptide, allowing selective cleavage of the peptide backbone. Due to their steric bulk, carbohydrates directly bound to the amino acid that is recognized by the protease can interfere with this binding, reducing or completely eliminating proteolysis by that enzyme. Carbohydrate placement on proximal or adjacent amino acids may lead to reduced availability of the targeted peptide for protease binding (such as the binding of trypsin to Lys or Arg residues). Glycosylation at or near the N-terminus (NT) can inhibit the activity of dipeptidyl peptidase IV (DPP-IV) (Werle and Bernkop-Schnurch, 2006), but because in GPCR-agonist peptides the NT is generally associated with the “message” segment of a peptide and receptor activation, this can lead to a concurrent decrease in activity. Modifications such as inversion of the second amino acid (from *L* to *D*) can mitigate DPP-IV activity.

Helical peptide conformations, as well as other tertiary structures, impede the binding of some proteases. Some early studies suggested that glycosylation of peptides may inhibit helix formation in aqueous environments (Bertozzi et al., 1992). Early work by Polt and coworkers to address this concern suggested that some disruption of helical systems can occur; although much of their model peptide was locked as a helix via a disulfide bridge, the amino acid closest to the carbohydrate appeared less helical than its non-glycosylated analog (Kriss et al., 2000). Later, Polt and coworkers demonstrated that helicity of a peptide is more strongly associated with the peptide sequence than with the presence or absence of a carbohydrate (Palian et al., 2001). In that experiment, a peptide known to evidence helicity in aqueous conditions was modified with a serine residue, which had previously been shown to reduce helical character (Merutka and Stellwagen, 1990), and with a serine α-mannoside. NMR and CD data collected on the peptides showed minimal variations in helicity between the two peptides, although both were less helical than the unmodified peptide.

Subsequent experiments have yielded opposing views on the disruption of helicity by carbohydrates. Glycosylation of a series of endorphins showed that the measured amount of helicity of a peptide can be impacted by the peptide's glycosylation state (Li et al., 2012). In that study, the non-glycosylated peptide is helical, but the addition of a monosaccharide disrupts the helicity, and increasing the carbohydrate size to a disaccharide leads to further decreases in helicity (although the glycopeptides remain active). Other work showed that glycosylation appears to only have a minimal effect on the helical nature of α-helical (Palian et al., 2003; Li et al., 2014b) and coiled coil glycopeptides (Falenski et al., 2010), and in some cases may stabilize the peptide's tertiary structure (Andersson et al., 1998). Therefore, when a helical system is required in a peptide, studies must be made on a case-by-case basis to determine if glycosylation will affect the peptide secondary structure. Jensen and coworkers took into account these considerations while designing and running a targeted “glycoscan” (substituting native peptides for either a serine glucoside or serine galactoside) of analogs of PYY3-36, a peptide implicated as a potential therapeutic for obesity (Pedersen et al., 2010). The researchers noted that selective installation of carbohydrates increased the half-life of their peptides against two proteases, while affecting helicity (both positively and negatively) and receptor affinity in a site-dependent manner.

### Distribution, metabolism, and pharmacokinetic effects

Autocrine and paracrine peptides are produced near their related receptors, and all are quickly recycled, regardless of whether or not receptor activation occurs. Endocrine peptides, which travel via the bloodstream have longer half-lives, but are also susceptible to enzymatic degradation mechanisms. In the context of our highly regulated bodies, many endogenous peptides are produced nearby to their related receptors (D'Souza et al., 2004). Any peptides that do not perform their designed function are quickly recycled by various enzymatic mechanisms, Modi (1994) preventing peptides from interacting with receptors in other areas. For peptide drugs, this proves a sizeable barrier, as the ideal pathways for introducing peptides to the body, via oral or nasal administration, often leave them far removed from their desired locations of activity (Fosgerau and Hoffmann, 2015). Many peptides have serum half-lives *in vivo* similar to the time it takes a blood cell to circulate through the bloodstream (1–1.5 min), reducing the chance that an exogenous peptide will ever reach its destination. Therefore, prolonging the serum half-life is imperative for improving the chances of the peptide reaching its desired target. For oral administration, most peptides do not have dedicated mechanisms for crossing membranes, a number of which are present along the pathway from mouth to brain.

**Figure 1:**
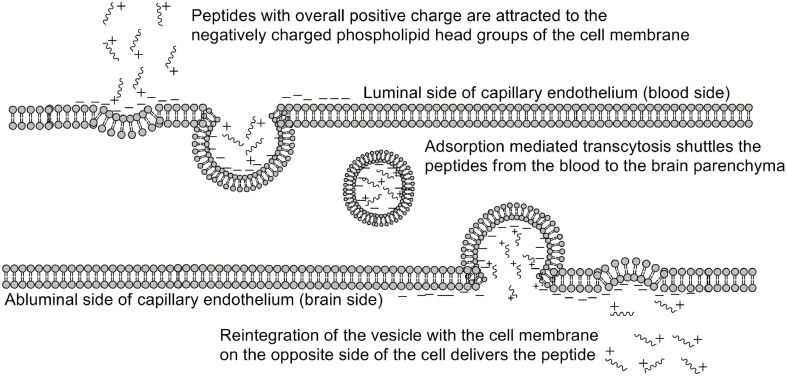
**Adsorptive mediated transcytosis as pathway for BBB penetration**. Positively charged peptides in the bloodstream (1) associate with the negatively charged membrane surface, thereby inducing membrane invagination and vesicle formation. The peptide-containing vesicle (2) then enters the intracellular space, and may reintegrate with the CNS-side of the membrane (3), releasing the peptides. Adapted from personal communications with Dr. Bobbi Anglin.

**Figure 2:**
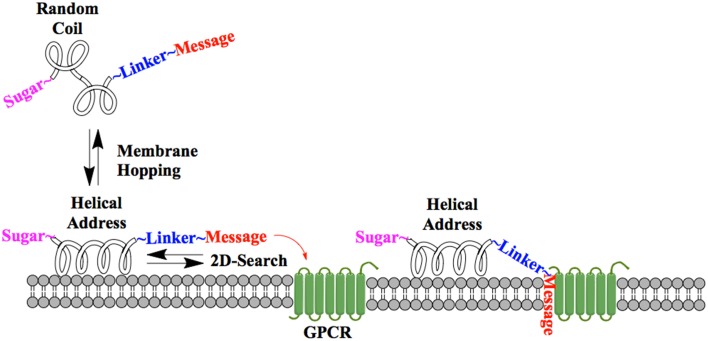
**Peptide-membrane interaction**. In the presence of a membrane, a glycopeptide adopts a helical conformation and undergoes a two-dimensional “search” of the membrane exterior. If the message segment interacts with a matched receptor, binding and activation occurs.

**Figure 3:**
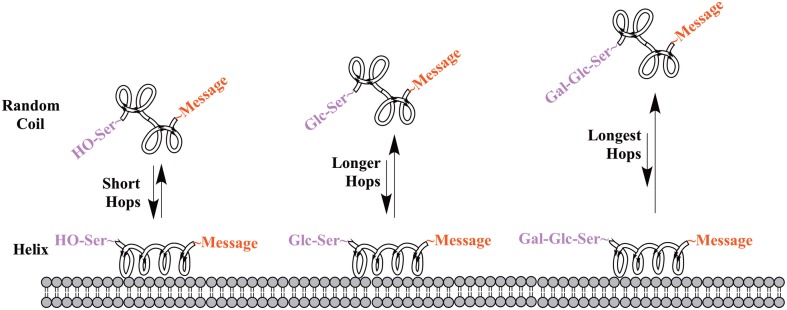
**Proposed mechanism of membrane “hopping.”** In the CNS, a peptide will be present in equilibrium between two primary conformations: a random coil in aqueous environments, and a helix on a membrane surface. This equilibrium is governed by the peptide's ampipathicity: the proportion of the peptide that is hydrophilic vs. that which is hydrophobic. Theoretically, a glycopeptide will have a higher affinity for the aqueous environment than its non-glycosylated analog, leading to reduced time spent on the membrane surface. This is hypothesized to lead to longer “hops,” presumably allowing a glycopeptide to interrogate more of a membrane surface and find receptors.

One of the earliest indications that glycosylating compounds targeted at the CNS may improve their pharmacokinetics was the observation that morphine-6-glucuronide and morphine-3-glucuronide improve the *in vivo* bioavailability of morphine (as a μ-opioid receptor agonist and antagonist, respectively) (Osborne et al., 1988; Mulder, 1992). Since then, multiple groups have shown that glycosylation of a peptide increases membrane penetration, both in the intestinal tract (Nomoto et al., 1998), and the blood-brain barrier (BBB) (Poduslo and Curran, 1994; Polt et al., 1994; Dhanasekaran and Polt, 2005; Egleton et al., 2005; Otvos et al., 2008). It had been initially hypothesized that glycosylation of peptides increases BBB penetration by exploiting carbohydrate transporters such as glucose transporter GLUT-1 (Polt et al., 1994), but this was later shown to be incorrect (Li et al., 2012). In fact, Rocchi and coworkers observed that their galactose-modified peptide entered the neurovascular unit twice as rapidly as the glucose analog. (Negri et al., 1998) Polycationic peptides may cross via adsorptive transcytosis, (Figure 1) in which the positively charged peptide associates with the negatively charged head groups of a membrane, a vesicle is formed around it, and the peptide is delivered to the other side of the membrane (Herve et al., 2008). It is also hypothesized that peptides find transmembrane receptors by performing a two-dimensional “search” of the membrane. As shown in Figure 2, peptides that exist in a random coil in aqueous environs adopt a helical conformation upon association with a membrane, and subsequently “search” for a matched receptor. As equilibrium exists for a peptide between its presence as either a membrane-bound helix or a random coil, modulation of amphipathicity may affect the amount of time a peptide spends in each state. This can be altered through the addition of a highly hydrophilic moiety such as a carbohydrate to a peptide (which trend hydrophobic), and may improve a peptide's affinity for the aqueous environment than its non-glycosylated analog. In turn, this may allow a glycopeptide to interrogate a larger membrane surface are, and increase the likelihood of finding a receptor (Li et al., 2012), as seen in Figure 3.

**Figure 4:**
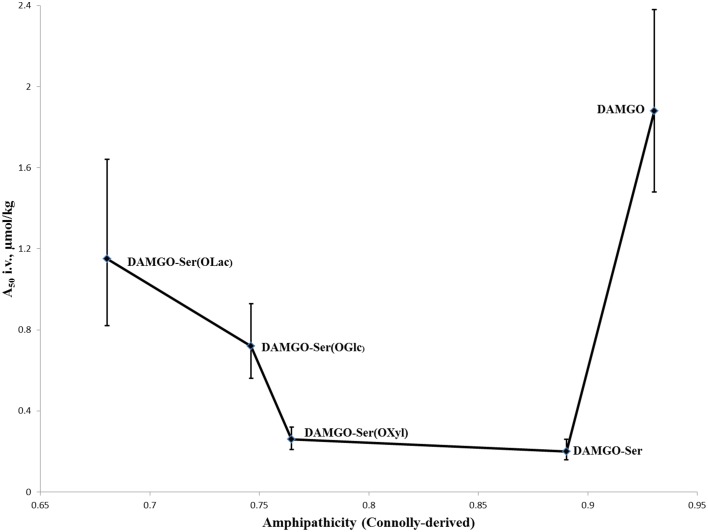
***I.v*. activity of DAMGO analogs vs. ampipathicity from Lowery et al. (2007)**. A series of DAMGO analogs [in order of increasing hydrophilicity: DAMGO, DAMGO-Ser(OH), DAMGO-Ser(OXyl), DAMGO-Ser(OGlc), DAMGO-Ser(OLac)] were tested for *i.v*. MOR activity using mouse tail-flick tests (Lowery et al., 2007). It was observed that initial increases to hydrophilicity significantly improved activity, but subsequent increases led to a decrease. This is believed to be the result of the peptides spending a preponderance of time in the aqueous phase, providing reduced opportunities for MOR binding and activation.

Justification for increased bioavailability to the brain was proposed by Polt and coworkers through what they term “biousian” (*bi*—two, and *ousia*—essence) behavior: essentially a measure of peptide amphipathicity, generally as the ratio of the hydrophilic glycoside to the relatively hydrophobic peptide backbone (Egleton et al., 2005). This hypothesis was further encouraged experimentally by observations that *i.v*. activity of modified glycopeptides adopts roughly a “U-shape” (Figure 4) when plotted against computationally-derived Connolly surface areas (e^−Awater/Alipid^) amphipathicity (Lowery et al., 2007). In short, appending a small carbohydrate to a neuropeptide decreased the *i.v*. A_50_-value, but as the size of the carbohydrate increased the A_50_-value did as well. This suggests that addition of a small carbohydrate allowed the peptide to probe a larger surface area for receptors through “hopping,” but larger carbohydrates displayed too strong a preference for the aqueous environment, and not the membrane where receptors are located.

Finally, it is worth noting that some animals use glycosylated, neurotoxic peptides as a self-defense mechanism. Many cone snails use a glycosylated conotoxins to paralyze attackers (Gerwig et al., 2013). Olivera and coworkers (Craig et al., 1999), among others, synthesized non-glycosylated versions of a number of these peptides, and found that although the non-glycosylated versions showed improved neurotensin receptor binding affinity compared with the native, glycosylated peptides, the native peptides still showed a greater than 10-fold increase in *i.v*. activity. This strongly suggests that the decreased enzymatic degradation afforded by the glycosylated peptide is significant enough that evolutionarily developing the glycopeptide was preferred over its simpler, non-glycosylated analog (Craig et al., 2001).

## Synthetic methods of o-linked carbohydrate incorporation

O-linked carbohydrates are of particular interest in glycopeptide development due to the frequency with which amino acids that have hydroxyl-terminated side chains appear in living systems. Synthetic glycopeptides are commonly prepared using solid-phase peptide synthesis (SPPS) fluorenylmethyloxycarbonyl (Fmoc) chemistry methodology (Polt et al., 1992; Lefever et al., 2012). Sugars including glucose, lactose, galactose, mannose, xylose, glucosamine, and galactosamine have been affixed to the amino acids serine, threonine, and hydroxyproline (Rodriguez et al., 1989). It has been reported that glycosylated amino acids are more likely to epimerize during SPPS (Zhang et al., 2012b) but modifications including reagent selection and substitution of similar amino acids, for instance Thr for Ser, can reduce this undesirable side reaction (Zhang et al., 2012a). Li and coworkers have also shown that ionic liquids can be used in place of resin during glycopeptide syntheses (Li et al., 2014a).

Early O-glycosylation of amino acids, reviewed in a number of places, Meldal and St Hilaire (1997) and Herzner et al. (2000) typically used a Koenigs-Knorr approach to form the glycosyl bond. In this methodology, strong Lewis acids are used to displace a previously installed leaving group, typically a halide, at the anomeric position to form an oxonium. The nucleophile, in most cases the free hydroxyl of an Fmoc-protected amino acid, then forms a bond at carbon-1, leading to either the α or β anomer. By incorporating a directing group on the adjacent carbohydrate hydroxyl, bias toward either α- or β-O-linked amino acids can be achieved. Other methods, including those with Schiff base protected amino acids (Polt et al., 1992; Mitchell et al., 2001) or using SnCl_3_(ClO_4_) to produce the linkage between an acetylated carbohydrate and TMS-protected Fmoc-amino acid (Broddefalk et al., 1994) have also been reported as methodologies to improve α/β selectivity or provide more amenable reaction conditions.

In our lab, O-linkages to amino acids are performed directly between Fmoc-protected amino acids with unprotected side chains and peracetylated sugars. Use of peracetylated sugars with amino acids allows their incorporation at any location within the peptide and time during the synthesis. The acetates can be removed orthogonally at the completion of the synthesis using hydrazine or Zemplén conditions prior to cleaving the glycopeptide from resin using standard TFA-based cleavage conditions. A weak Lewis acid, such as InBr_3_ or Bi(OTf)_3_, is used catalytically to drive the reaction to completion within a couple hours (Lefever et al., 2012).

Toth and coworkers have reported the use of glycosyltransferases to enzymatically append a carbohydrate to previously prepared lactosylated Leu-enkephalin (Simerska et al., 2013; Christie et al., 2014). This preparation has led to compounds that ultimately improve the binding of the enkephalin analogs to their desired target, the asialoglycoprotein receptor.

## Development and applications of neurologically active glycopeptides

As referenced earlier, glycoantibiotics such as vancomycin, bleomycin A_2_, and the “Sushi” peptides (Ding et al., 2008) have been used worldwide to treat millions. These examples show that using glycopeptides as drugs can successfully overcome many of the obstacles that prevent widespread use of non-glycosylated peptides.

### Opioids—enkephalins

Some of the first chemistry to validate the hypothesis that glycosylation of a neuropeptide improves its pharmacokinetic profile was through the modification of enkephalins (Polt et al., 1992, 1994). Enkephalins are endogenous pentapeptides that are partially selective for the δ-opioid receptor (DOR). Polt and coworkers modified an enkephalin analog, [D-Pen^2,5^]enkephalin (DPDPE) (Polt et al., 1994). It was observed that glycosylation significantly improved the *intraperitoneal* (*i.p*.) antinociceptive effects of the peptides when compared to those of the non-glycosylated version. Further work to expand the number of amino acids and carbohydrates, including both mono- and disaccharides, that could be introduced into synthetic peptides gave evidence of the broad scope of modifications possible based on this technology (Mitchell et al., 2001). Concurrently, modifications to the native forms of enkephalin, Leu-enkephalin (Bilsky et al., 2000), and Met-enkephalin (Egleton et al., 2000), were also being performed. Similar improvements to antinociception were observed, and additional work demonstrated that these particular glycopeptides decreased the drug dependence commonly associated with opioid receptor agonists, while improving their central neurological bioavailability. Later, Toth and coworkers reported that glycosylations of Leu-enkephalin yielded increased binding to the asialoglycoprotein receptor, a receptor endogenously targeted by galactose-terminated glycoproteins (Simerska et al., 2013; Christie et al., 2014).

### Opioids—endorphins

To a lesser extent, research has also been performed on glycosylated endorphins, 31 amino acids endogenous μ-opioid receptor (MOR) antagonists. One of these, β-endorphin, shares the same five amino acids at the N-terminus (NT) as Met-enkephalin, and was targeted for further studies. Due to its length, β-endorphin was modified to test the necessity of helicity for receptor binding, and determining how glycosylation either assists or hinders helix formation (Li et al., 2012, 2014b). Various spacers, including γ-aminobutyric acid (GABA) and δ-aminovaleric acid (DAVA), as well as unnatural amino acids such as 2-aminoisobutyric acid (Aib) were introduced to allow localized helicity. These studies confirmed that amphipathicity is crucial to membrane association of peptides, and that glycosylation provides a very important variation to amphipathicity, presumably through an increase to its hydrophilic nature. As the authors state, the amphipathicity provided by the carbohydrate of a glycopeptide “appears essential for the effective transport of these larger peptides across the BBB” (Li et al., 2012).

### Other CNS-active peptides

Further modifications to enkephalins and endorphins led to mixed MOR/DOR agonists, such as MMP-2200 (Palian et al., 2003). Since its publication, MMP-2200 has shown to be active both as an antinociceptive via mixed MOR/DOR opioid agonism with activity in the μmol/kg range (Elmagbari et al., 2004; Lowery et al., 2011) and in preclinical Parkinson's disease models by blocking induced hyper-kinesia (Yue et al., 2011). The authors attribute the latter activity to reducing the downstream effects of various dopamine agonists in their models.

Rocchi and coworkers performed studies with glycosylated dermorphin analogs (Negri et al., 1998, 1999). They noted that the addition of carbohydrates afforded improved DMPKs via reduced susceptibility to enzymatic degradation and improved passage across the BBB. In one case they observed that despite displaying lower affinity for the DOR than native dermorphin, a galactosylated analog afforded much better *in vivo* antinociception when administered peripherally.

The potent MOR-selective agonist DAMGO [Tyr-ala-Gly-(NMe)Phe-Glyol] was modified to test the membrane hopping hypothesis introduced above (Lowery et al., 2007). The antinociceptive activity of DAMGO was compared with a series of increasingly hydrophilic glycosylated derivatives (Figure 4). The researchers determined that the glycosylated DAMGO analogs maintained their MOR selectivity, and after *i.v*. administration the level of antinociception produced a “U-shaped” curve, where the A_50_ decreased upon the addition of a small carbohydrate, but as the size of the carbohydrate increased, so too did the A_50_. Presumably this activity is due to the latter molecules' propensity to remain in aqueous solution as opposed to in a membrane-bound state where receptor activation is possible.

Mosberg and coworkers developed a combined cyclic tetrapeptide that provided potent MOR agonist/DOR antagonist (Purington et al., 2011) but like many peptides, it suffered from poor CNS bioavailability. Upon appendage of a serine glucoside to the C-terminus, their peptide exhibited *i.v*. antinociceptive activity similar to that of morphine, without the development of acute tolerance that is a major side effect of morphine use.

The secretin family of peptides, including secretin, glucagon, pituitary adenylate cyclase-activating polypeptide (PACAP), and vasoactive intestinal peptide (VIP), are a series of closely related peptides and receptors that have a wide range of central and peripheral nervous system activities. Recently, VIP and PACAP have been identified as potential modulators of CNS immune system response (Waschek, 2013) which has ramifications for neurodegenerative diseases such as Alzheimer's disease, Parkinson's disease and chronic traumatic encephalopathy, and for traumatic brain injuries and strokes. However, these peptides do not cross the BBB in appreciable quantities. Rocchi and coworkers performed a novel “glyco-scan” of VIP to increase its metabolic stability (Dangoor et al., 2008). They discovered that all glycosylated peptides showed some decrease in receptor affinity. Only one of their modified peptides showed a significant increase in stability toward trypsin. To date there are no published reports of *in vivo* tests of glycosylated secretins, although another group (Bourgault et al., 2011) has also recognized the potential for glycosylation to improve the neuroprotective behavior of PACAP.

## Future directions and applications

With the significant headway scientists have made in the field, the future is bright for glycopeptides. Recently glycopeptides have been incorporated into various types of nanoparticles (NPs). It was reported that MMP-2200 had been successfully conjugated to poly(lactic-*co*-glycolic acid) (PLGA) NPs (Tosi et al., 2011, 2014). Interestingly, these NPs increased BBB penetration of the peptide, likely through a combination of protecting the peptides from proteases in the bloodstream and along membranes, and improving the rate of endocytosis across the BBB membrane. Another group (Lotfipour et al., 2014) successfully loaded vancomycin into PLGA NPs and demonstrated that although the antibiotic loses some of its *in vitro* antimicrobial activity, it is slowly released from NPs, perhaps providing a mechanism maintain therapeutic levels of a glycopeptide over time. Other work has involved the tethering of glycopeptides to gold NPs (Parry et al., 2013; Tavernaro et al., 2015). In one instance, tethered MUC1-glycopeptide antigens were shown to allow the glycopeptides to bypass most of the immune system and enzymatic degradation pathways, and represents an attractive future pathway for drug delivery.

Finally, recent developments of analytical techniques have led to new methodologies for measuring BBB penetration *in vivo*, via microdialysis. Kennedy and coworkers demonstrated that after *i.p*. co-injection of MMP-2200 and Leu-enkephalin, they were able to collect dialysate from the dorsolateral striatum that showed the presence of these peptides in the nM range within 10 min (Mabrouk et al., 2012). Use of microdialysis for BBB penetration analysis is a very attractive option for two reasons. First, it offers the ability to test multiple compounds concurrently, in a single living system. Second, researchers may perform multiple tests before sacrificing the animal, leading to fewer rodents necessary to validate each new potential therapeutic.

Taken together, the pioneering glycopeptide work described herein combined with the diverse and imaginative applications currently undergoing study in the field show that glycopeptide research has diverse opportunities for continued advances in drug discovery.

### Conflict of interest statement

We have several patents in this area, and have founded a Delaware-registered company, Bioussian Biosystems, Inc., that has a delta-opiate selective agonist for chronic pain. That work is not mentioned in the manuscript. The authors declare that the research was conducted in the absence of any commercial or financial relationships that could be construed as a potential conflict of interest.
